# Improved Enumeration of Weakly Fluorescent CD4+ T-lymphocytes by Confining Cells in a Spinning Sample Cartridge with a Helical Minichannel

**DOI:** 10.3390/mi11060618

**Published:** 2020-06-25

**Authors:** Subin Kim, Jakir Hossain Imran, Mohiuddin Khan Shourav, Jung Kyung Kim

**Affiliations:** 1 Department of Mechanical Engineering, Graduate School, Kookmin University, 77 Jeongneung-ro, Seongbuk-gu, Seoul 02707, Korea; ksbyks@naver.com (S.K.); khan@kookmin.ac.kr (M.K.S.); 2 Department of Integrative Biomedical Science and Engineering, Graduate School, Kookmin University, 77 Jeongneung-ro, Seongbuk-gu, Seoul 02707, Korea; jhimran11@kookmin.ac.kr; 3 School of Mechanical Engineering, Kookmin University, 77 Jeongneung-ro, Seongbuk-gu, Seoul 02707, Korea

**Keywords:** helical minichannel, spinning, confinement, particle migration, CD4, cell counting

## Abstract

The CD4 (cluster of differentiation 4) counting method is used to measure the number of CD4+ T-lymphocytes per microliter of blood and to evaluate the timing of the initiation of antiretroviral therapy as well as the effectiveness of treatment in patients with human immunodeficiency virus. We developed a three-dimensional helical minichannel-based sample cartridge in which a thread-like microgroove formed in the cylindrical surface and configured a particle-positioning and imaging system equipped with a single DC (direct current) motor that can be controlled by a smartphone application. Confinement and enrichment of CD4 cells within a sharp focal depth along the helical minichannel is accomplished by spinning the cylindrical sample cartridge at high speed before acquiring cell images and thus CD4+ cells with weak fluorescence intensity can be detected even in a channel much deeper than existing two-dimensional flat chambers without an autofocusing module. By detecting more cells in a larger sample volume, the accuracy of the CD4 cell count is improved by a factor of 5.8 with a channel of 500 μm depth and the precision is enhanced by a factor of 1.5 with a coefficient of variation of 2.6%.

## 1. Introduction

The human immunodeficiency virus (HIV) causes infection and acquired immunodeficiency syndrome (AIDS) and destroy CD4+ T-lymphocytes in the blood [1]. These T-helper cells expressing CD4 (cluster of differentiation 4) molecules on their surface play a crucial role in immune regulation, protecting the human immune system. As a result, the number of CD4 cells in such patients decreases gradually. A healthy person has more than 500 CD4 cells per one microliter of blood, whereas an HIV-infected patient has considerably fewer CD4 cells. The World Health Organization (WHO) recommends that adults with less than 350 CD4 cells/µL should be treated with antiretroviral therapy (ART) [2,3,4,5,6,7,8]. In developing countries, HIV/AIDS is currently one of the major causes of death and more than 95% of these deaths are caused by the lack of rapid diagnostic systems that can be used in resource-constrained regions [9,10].

Early diagnosis of HIV infection is considered potentially important from the viewpoint of public health and opportunity for treatment [11]. Therefore, point-of-care (POC) systems need to provide timely diagnosis. Studies have predicted the emergence of microfluidic devices as next-generation POC devices that can be integrated with reliable and accurate devices for various biomedical applications [12,13,14]. A review of emerging technologies for POC T-lymphocyte counting highlights technological developments in CD4 cell counting [15]. Emerging technologies incorporate the advantages of POC and commercial CD4 counting technologies, such as flow cytometry and automated imaging cytometry [16,17].

BD FACSCount^TM^ (BD Biosciences, San Jose, CA, USA) is a single-platform benchtop flow cytometer to enumerate the absolute number of CD4 cells and %CD4 values, where cell concentrations are calculated by adding fluorescent beads at a known concentration, allowing the volume-per-bead-detected to be calculated. However, this system still requires a skilled operator and regular maintenance and QC using calibration beads, thus lowering the test throughput. Partec GmbH (Görlitz, Germany) have launched CyFlow^®^ miniPOC, which can measure absolute CD4+ counts and %CD4, making it useful for monitoring both adults and infants. Pima^TM^ Analyzer (Alere Inc., Waltham, MA, USA)—equipped with an LED (light-emitting diode) illuminator and multicolor CCD (charge-coupled device)—analyzes static images of fluorescently-labeled CD4 and CD3 cells in a whole blood sample. This instrument is a fixed-volume imaging cytometer that can generate a CD4+ count, but not a %CD4 readout, limiting this system for use for infant HIV monitoring [15].

Most of the commercially available image-based CD4 test devices employ a flat shallow sample chamber, which ensures the detection of whole target cells positioned within the optical depth of field [18]. The low-profile sample chamber, however, may result in an erroneous absolute CD4 cell count with a large coefficient of variation, because the probe volume is not sufficient for statistical analysis unless a motorized stage is used synchronously with a camera [19,20]. To overcome this limitation, we previously proposed an unconventional sample cartridge with a screw thread-like microgroove formed in a cylindrical surface and built an in-house-developed florescence imaging setup with a DC (direct current) motor controlled wirelessly by a smartphone application [21,22]. An externally threaded section of the cylindrical sample cartridge was covered with an optically transparent adhesive tape to form a helical minichannel of depth 100 μm and width 600 μm and images of the microparticles in the circumferential minichannel were acquired through a fixed objective lens by rotating the cartridge stepwise using a nut–bolt mechanism. This simplified scanning of the sample by rotating the cartridge with one DC motor rather than translating an *xy*-stage, eliminates one motor.

Although the performance of CD4 count with our device was satisfactory, the accuracy was inferior to that of existing instruments. In this study, we propose a method to enhance the detection efficiency by confining cells within a focal plane by spinning the cartridge at high speed without limiting the channel depth and sample volume and without using an autofocusing module. We performed theoretical and experimental parametric studies to determine the effect of angular speed and channel depth on the position-dependent velocity of particle migration in the spinning helical minichannel. Subsequently, a feasibility study was conducted to demonstrate the improvement in accuracy and precision of the CD4 cell count by confining CD4 T-lymphocytes that emit a weak fluorescence signal in the deep channel.

## 2. Materials and Methods

### 2.1. Experimental Setup

**Figure 1:**
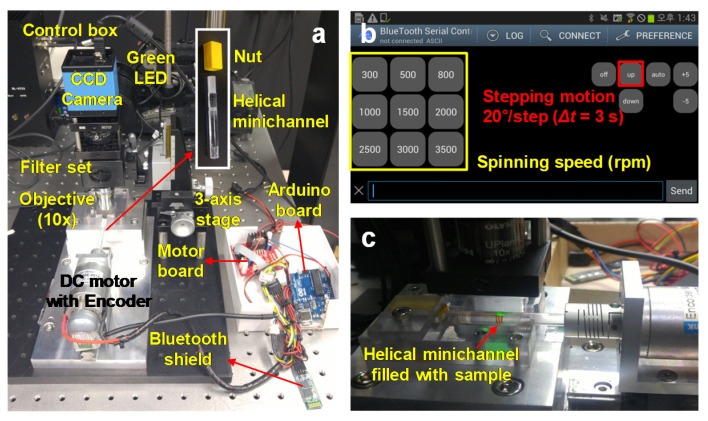
(**a**) Two-color-fluorescence imaging configuration with a Bluetooth-controlled DC (direct current) motor system; (**b**) smartphone application for motor control with an Arduino board; (**c**) helical minichannel filled with blood sample during acquisition of fluorescent cell images for counting.

**Figure 2:**
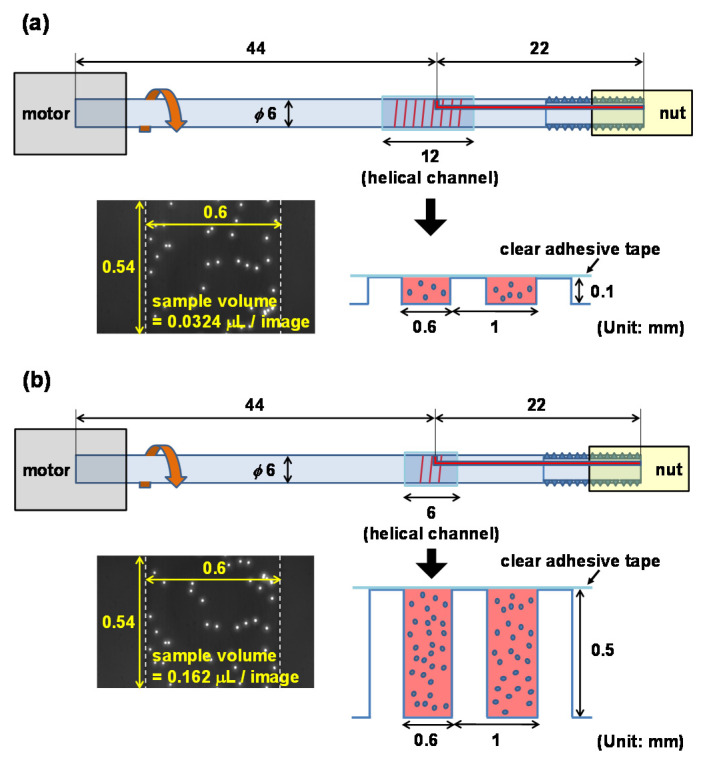
Schematic of the sample cartridge and the cross section of the helical minichannel with depths of (**a**) 100 μm and (**b**) 500 μm.

The helical system used herein comprised a two-color fluorescence imaging setup with a Bluetooth-controlled DC motor system, as shown in Figure 1a. For spinning, the helical channel was attached to a DC motor with an encoder for improving control of the spin speed. The DC motor was controlled by an Arduino board with a Bluetooth shield so that the spin speed and time could be controlled over a Bluetooth connection using a smartphone, as shown in Figure 1b. Camera focus was adjusted from above to observe particle position and confinement state. The particles were visualized horizontally using a 10× objective lens (0.3 NA) and a CCD camera (DMK23U445, The Imaging Source, Bremen, Germany) at a resolution of 1280 × 960 pixels. A camera synchronized with the motor obtained multiple images consecutively whenever the motor was rotated by 20°. Figure 1c shows the custom-made helical minichannel sample cartridge; its dimensions, cross-section; details are shown in Figure 2. The length and diameter of the cartridge are 66 mm and 6 mm, respectively. The channel width is 600 μm and the channel depth is 100 μm with 12 turns (Figure 2a) or 500 μm with 3 turns (Figure 2b). A screw thread covered by a transparent adhesive film forms a helical minichannel, so that the sample solution can be introduced into the channel through a hole at one end of the channel using a syringe.

### 2.2. Sample Preparation

To determine particle confinement in the helical minichannel, 10% fluorescent beads (F8833, Invitrogen, Carlsbad, CA, USA) measuring 10 μm in diameter and having a density of 1.06-g/mL were used to simulate the CD4 T-lymphocytes. The viscosities and densities of the media were controlled by adding glycerol solution to distilled water. To ensure that the composition of the media was similar to that of blood, 10% glycerol solution, which has a density similar to that of plasma (1.0225 g/mL), was used. To confirm confinement of the blood cells, blood was incubated with a fluorescent-labeled CD4-PE antibody (ab18282, Abcam, Cambridge, England) to stain the CD4 T-lymphocytes through the antigen-antibody reaction. To lyse the red blood cells, 200 µL of RBC lysis buffer and 100 µL of blood were mixed and incubated to react at room temperature for 15 min. After reacting, 10 µL of antibody was added to the blood sample in which RBC was dissolved and the mixture was reacted at room temperature for 15 min. Venous blood samples were collected from human volunteers with permission from the institutional review board (KMU-201412-BR-043).

### 2.3. Theoretical Background

**Figure 3:**
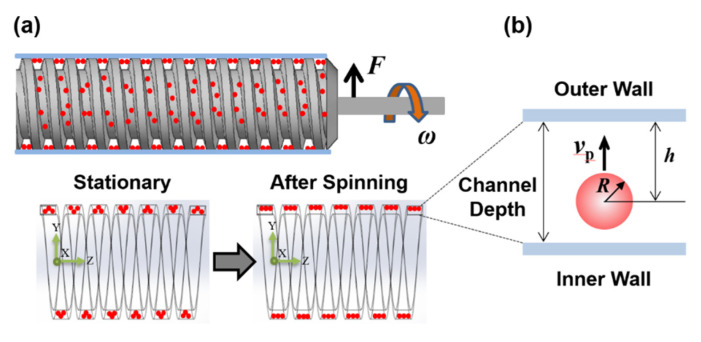
(**a**) Schematic of particle confinement in a helical minichannel after spinning the sample cartridge; (**b**) schematic of a particle moving toward the outer wall during spinning.

In industry and the laboratory, the centrifuge is used to separate the components of a mixture by centrifugal force. The high-density components of a mixture move away from the axis of rotation of the centrifuge and the low-density components move toward the axis, resulting in separation based on the density difference. We applied this separation principle to the helical minichannel-based sample cartridge using centrifugal force as illustrated in Figure 3. Three body forces act on particles in the helical minichannel; centrifugal force (*F*_c_), buoyancy force (*F*_b_) and drag force (*F*_d_). Centrifugal force is generated by rotation of the cartridge and buoyancy and drag forces are generated when the particles pass through a fluid. The centrifugal force acting on the particles is partially reduced owing to buoyancy and drag during centrifugation. Therefore, all forces acting on the particles are as follows.

(1) $$F_{c}=mr\omega^{2}=\rho_{p}\frac{\pi D^{3}}{6}\omega^{2}r$$

(2) $$F_{b}=\rho \frac{\pi D^{3}}{6}g$$

(3) $$F_{d}=3\pi \mu D\nu_{p}\lambda^{\;}$$

where *m* is the mass of the particle, *r* is the radial position of the particle, *ω* is the rotation angular frequency of the cartridge, $\rho_{p}$ is the density of the particle, ρ is the density of the solution, μ is the viscosity of the solution, *D* is the diameter of the particle, g is the gravity and $\nu_{p}$ is the velocity of the particle. Moreover,  λ is a drag correction factor for effects occurring at the channel walls, for which we employed a 12th-order interpolation formula with 6 coefficients for axial drag [23]. The interpolation formula is given as follows:

(4) $$\lambda =\frac{\gamma_{⊥}}{\gamma_{0}}$$

(5) $$\gamma_{⊥}=\frac{\gamma_{0}}{1-\frac{9R}{8h}+\frac{R^{3}}{2h^{3}}-\frac{57R^{4}}{100h^{4}}+\frac{R^{5}}{5h^{5}}+\frac{7R^{11}}{200h^{11}}-\frac{R^{12}}{25h^{12}}}$$

where $\gamma_{⊥}$ is the axial drag coefficient, $\gamma_{0}$ is the Stokes drag coefficient, *R* is the particle radius and *h* is the distance between the particle center and the outer wall. The correction formula is used to determine the particle velocity when the particle moves toward the outer wall of the channel.

### 2.4. Particle Confinement

After loading the sample into the helical minichannel, the particle positions were compared before and after spinning the cartridge to confirm the effect of spinning on particle confinement. We used three spin speeds ranging from 1000 to 3000 rpm with 1000-rpm interval to confirm confinement and enrichment of the particles versus rotation time. We measured particle position by spinning the cartridge for 10–60 s in 10 s intervals and additionally for 90 and 120 s.

### 2.5. Data Acquisition and Image Analysis

Multiple images of the sample particles in the helical minichannel were obtained by the camera synchronized with the motor. To analyze the obtained images, the particles were checked using image analysis software (ImageJ, http://imagej.nih.gov/ij/). We checked the degree of blurring by analyzing the area of the particles. Thus, we could determine the depths of the particles. To compare the performance of particle detection and counting with and without spinning, images of fluorescent beads and CD4 cells were acquired and analyzed in ImageJ by adjusting the threshold from 80 to 255 to remove blurred particles. Further, the number of remaining particles was counted. The particle concentration was determined by counting the total number of particles within the given sample volume. For a channel with a depth of 500 μm, 0.162 μL of the sample with a width of 600 μm and length of 0.54 mm was analyzed per image. By acquiring multiple images, 8.7 μL of the sample can be analyzed in total.

## 3. Results

### 3.1. Theoretical Analysis

**Figure 4:**
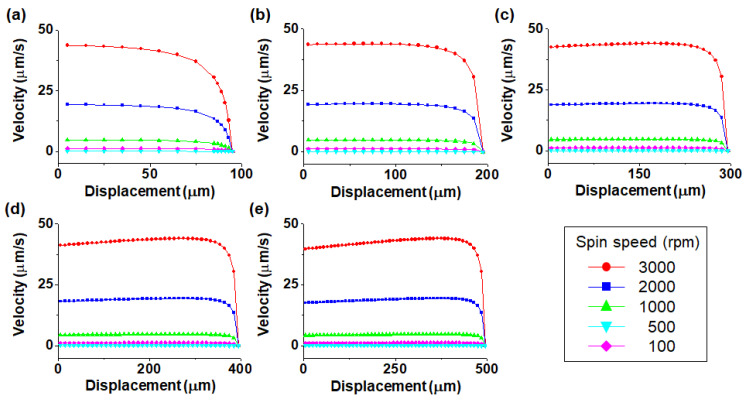
Plots of velocity–displacement as a function of channel depth for channel depths of (**a**) 100 μm, (**b**) 200 μm, (**c**) 300 μm, (**d**) 400 μm and (**e**) 500 μm.

Figure 4 shows the relationship between the particle velocity and displacement from the bottom to the top of the channel as a function of spin speed. The channel depth was varied from 100 to 500 μm in 100 μm steps. For the channel depth of 100 μm, the particle velocity decreased gradually as the particles moved closer to the outside of the channel. However, in channels with channel depths greater than 200 μm, the particle velocity increased and then decreased after reaching a critical point. A threshold was observed at 115 μm below the top of the channel at which the speed decreased for channel depths greater than 200 μm. The reason for the rapid decrease in particle velocity in the proximity of the wall is that the centrifugal force acting on the particles is influenced by the wall of the channel.

**Figure 5:**
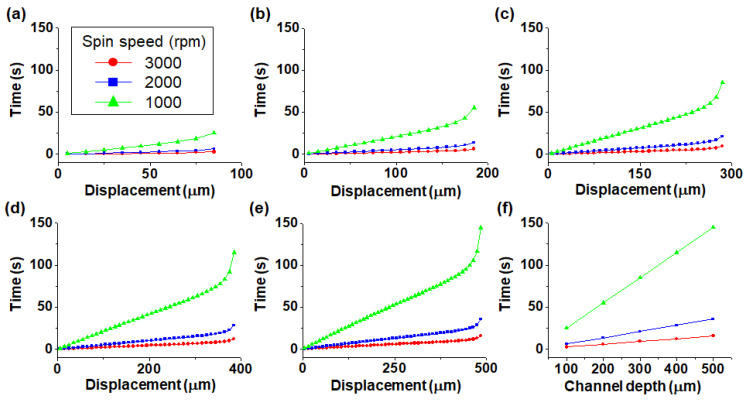
Plots of time–displacement for channel depths of (**a**) 100 μm, (**b**) 200 μm, (**c**) 300 μm, (**d**) 400 μm and (**e**) 500 μm; (**f**) Time taken by particles to reach the top of the channel depending on channel depth (h = 15 μm).

Figure 5 shows the relationship between the rotation time and displacement from the bottom to the top of the channel as a function of spin speed. Compared with the velocity plots shown in Figure 4, the graphs in Figure 5a–e show that the required rotation time increases gradually from the bottom and eventually increases sharply near the top where the velocity decreases abruptly. Further, the greater the channel depth, the greater is the required rotation time. The required rotation time for particle confinement according to channel depth can be obtained from Figure 5f. For instance, when a channel with a depth of 500 μm rotates at 1000 rpm, the rotation time required for the particles to be displaced 485 μm from the bottom of the channel is approximately 145 s.

### 3.2. Experimental Results

#### 3.2.1. Intensities of Fluorescent Beads and CD4 Cells

**Figure 6:**
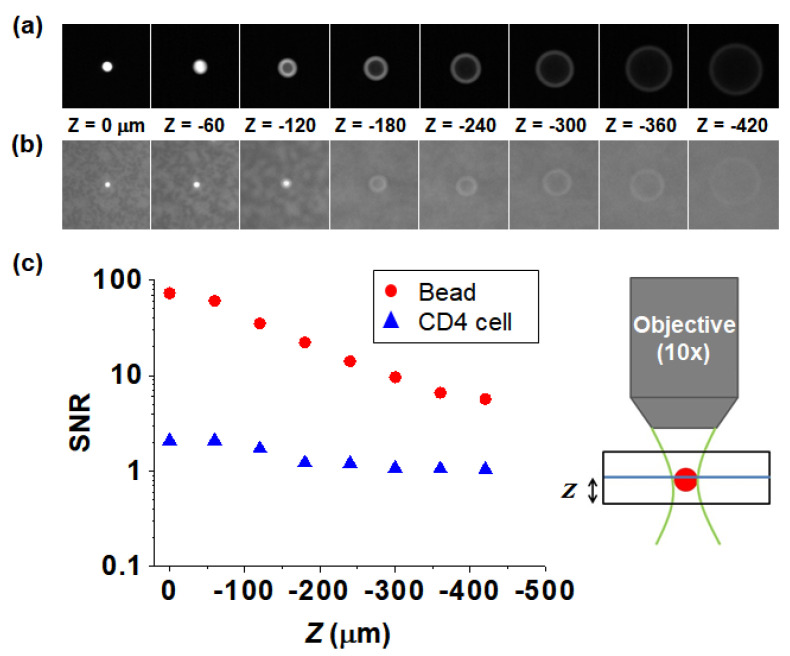
Sequence of particle and CD4 (cluster of differentiation 4) cell images taken at different z-axis offsets using a 10× objective lens; (**a**) 10-µm fluorescent bead; (**b**) fluorescently-labeled CD4+ T-lymphocyte; (**c**) comparison of the signal-to-noise ratios (SNRs) of the fluorescent bead and CD4 cell.

The molecules of the equivalent soluble fluorochrome (MESF) value are used for quantifying the fluorescence intensity of a stained sample. The MESF values of the fluorescent-labeled CD4 cells and the fluorescent beads were determined using calibration beads (Quantum^TM^ MESF Kits, Bangs Laboratories, Fishers, IN, USA) and found to be approximately 67,310 and greater than 291,866, respectively. This means that fluorescent beads can be detected efficiently even if they are not in focus; however, the fluorescence intensity of the CD4 cells is so weak that they are difficult to detect without tight focusing. Therefore, if the fluorescence intensity is weak, the effect of focus is increased. Figure 6a,b show images of a fluorescent bead and CD4 cell adhered to a coverslip, respectively, taken at different z-axis offsets for a 10× objective. Camera exposure times for the fluorescent-bead and CD4-cell images were 0.001 s and 0.5 s, respectively. Figure 6c shows the signal-to-noise ratio (SNR) as a function of the z-position obtained by dividing the average signal value of the respective sample by the value of the background signal. The SNR of the fluorescent beads is 5–72, which, despite the short exposure time, is much larger than the extremely low value of 1–2 of the CD4 cells. To detect CD4 cells with weak fluorescence intensity, it is necessary to use a low-profile channel that does not require focus adjustment. However, a shallow channel has a high flow resistance; moreover, the enumeration accuracy would decrease if such a configuration were employed, because the analysis is performed with a limited sample volume. A means to confine CD4 cells to the focal plane inside a deep containing channel, it would enable us to analyze a large sample volume by concentrating the cells without adjusting the focus across the cross section.

#### 3.2.2. Particle Confinement

**Figure 7:**
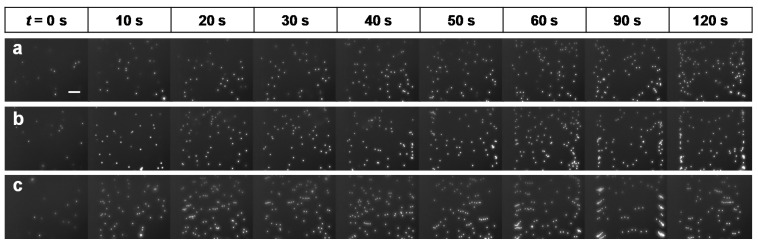
Particle confinement in the helical minichannel with 500 μm depth for 10-μm fluorescent beads suspended in 10% glycerol before (0 s) and after (10–120 s) spinning (**a**) at 1000 rpm, (**b**) at 2000 rpm and (**c**) at 3000 rpm. (scale bar = 0.1 mm).

Figure 7 shows particle confinement in the 500-μm-deep channel for a 10% glycerol sample before and after spinning from 0 to 120 s. Figure 7a shows that after filling the channel (stationary at 0 s) no particle confinement was obtained in the channel. When the channel was spun at 1000 rpm for 2 min, the particles moved to the top of the channel owing to centrifugal force; as a result, all particles were observed at the same z-position (the focus). Figure 7b shows a result similar to that obtained in the previous case, where all particles were scattered inside the channel before spinning; however, after the channel was spun at 2000 rpm for 40 s, the particles were confined to the same z-position, having been lifted to the desired position of the outer wall of the channel. Figure 7c shows that at 3000 rpm, particle confinement was achieved more quickly than it was at 1000 rpm or 2000 rpm. The elapsed time for confining particles decreased as the cartridge spin speed increased; however, at 3000 rpm, the particles agglomerated to form clusters. As shown in Figure 7, the elapsed time for confining particles depends on the spin speed.

#### 3.2.3. Particle Count

**Figure 8:**
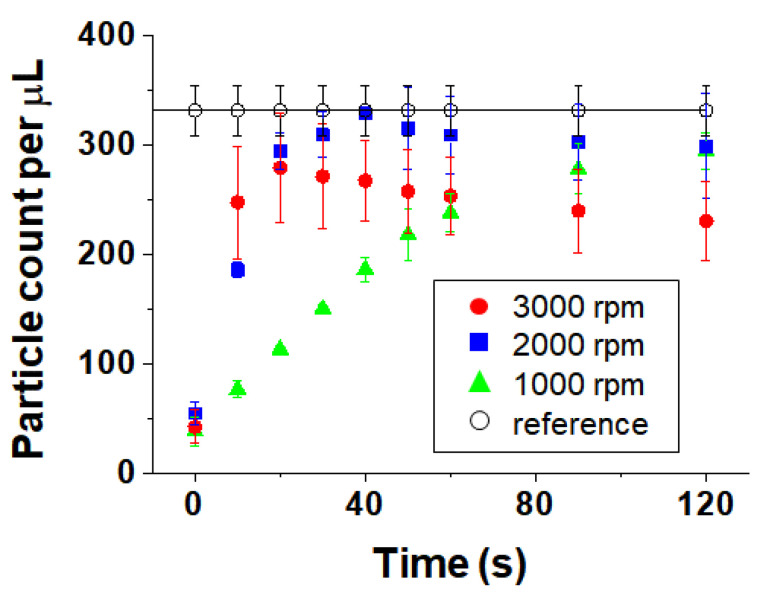
Particle counts depending on time at different spin speeds (1000, 2000 and 3000 rpm).

We analyzed particle confinement before and after spinning by detecting particles near the top of the helical minichannel and counted only focused particles. The blurred particles were found to be scattered at a depth of 500 μm in the channel. Figure 8 shows the particle counting results as a function of time at different spin speeds. The plot shows the effect of spinning on particle count accuracy. The reference value of 331 particles/µL was obtained by using the manual counting method with a hemocytometer chamber (C-Chip, INCYTO, Cheonan, Chungnam, South Korea). Before spinning the cartridge (at 0 s), the number of particles was considerably lower than the reference value. This means that the particles were randomly distributed throughout the channel and were not detected efficiently because of blurring.

After spinning for 10 s, the number of particles counted increased considerably compared with that before spinning. At the spin speed of 3000 rpm, the number of particles counted increased most rapidly. However, at the spin speed of 1000 rpm, the number of particles increased gradually up to 120 s, converging to a final number of particles similar to the reference value. At the spin speed of 2000 rpm, the number of particles increased gradually up to 40 s and was very similar to the reference value; however, further rotation reduced the particle count slightly because some particles agglomerated and could not be counted individually. At 3000 rpm, the particles moved through the channel the fastest, whereas the number of particles decreased as the rotation time increased because the particles agglomerated rapidly.

The higher the spin speed, the faster the initial particle confinement; however, the accompanying error range was large and particle counts were less accurate owing to particle agglomeration. Low spin speeds, nevertheless, are unsuitable for POC devices because the particles take a long time to move through the channel. Therefore, an optimized spin speed and time are required for particle confinement under centrifugal force, which depend on the density of the sample solution and particles. In the case of fluorescent particles in a 10% glycerol solution, after rotation at 2000 rpm for 40 s, the particles could be counted accurately without adjustment of optical focus.

### 3.3. Comparison between Theory and Experiment

Comparing the time at which particles were confined out of the channel at each spin speed with the theoretical values by using a 500-μm-deep channel, when the spin speed was 1000 rpm, at least 120 s was required for particle confinement, as determined experimentally, while the corresponding theoretical value was 143 s. At 2000 rpm, the experimental value was approximately 40 s and the theoretical value was 35 s. At 3000 rpm, the theoretical value was 15 s; however, an experimental value could not be obtained because the spin speed was too high and the particles agglomerated, leading to undercounting. In Figure 8, at 3000 rpm, the number of particles initially increases rapidly, and it is assumed that the particles would be well confined at approximately 20 s if there were no particle agglomeration. Thus, the experimentally observed and theoretical times required for particles to move outward according to the spin speed are similar in value and agree in trend.

### 3.4. CD4 Cell Count by Confinement

**Figure 9:**
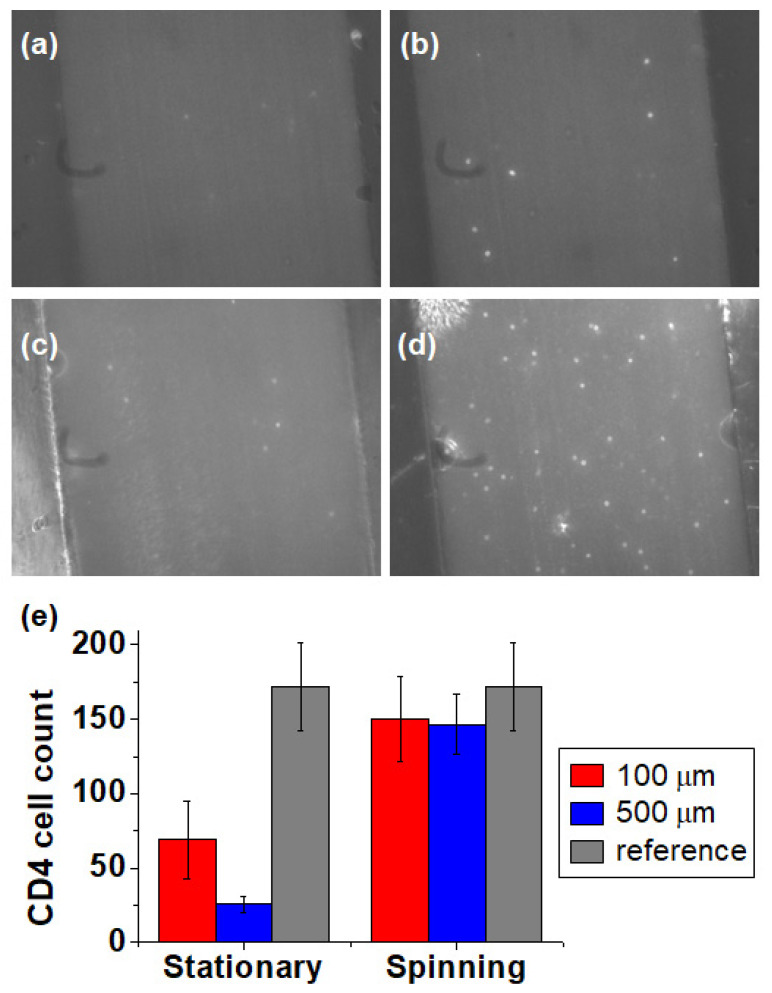
CD4 cell counts before and after spinning for different channel depths (100 and 500 µm); (**a**) before and (**b**) after spinning in a 100-µm-deep channel; (**c**) before and (**d**) after spinning in a 500-µm-deep channel; (**e**) comparison of the number of CD4 cells per µL before and after spinning in each channel with the reference value determined by manual counting using a C-Chip.

Based on particle confinement results obtained by spinning the cartridge, we applied the method to count CD4 cells using channels with depths of 100 µm and 500 µm. Figure 9a,c show images of CD4 cells before spinning the cartridges for depths of 100 µm and 500 µm. Some CD4 cells were detected at the focal plane, but many CD4 cells remained blurred or undetected. Therefore, CD4 cells are distributed in the height direction of the channel after they are loaded onto it. In Figure 9b,d, after spinning the cartridge at 2000 rpm for 1 min and 2 min, respectively, more CD4 cells are detected in the image. The number of CD4 cells detected per image in the 500-μm-deep channel was approximately 4.89 times the corresponding number for the 100-μm-deep channel. This indicates that cells can be confined and enriched at the focal plane through cell migration by centrifugal force and a larger number of cells detected corresponding to an increased sample volume. Figure 9e compares the CD4 cell count per μL of the 100-μm-deep and the 500-μm-deep channels before spinning (stationary) and after spinning. The cell concentration was measured by counting the total number of cells contained in the helical minichannel. For a channel with a depth of 500 μm, a width of 600 μm and length of 0.54 mm, 0.162 μL of the sample per image can be analyzed. By taking cell images along the channel, 8.7 μL of the sample can be analyzed in total. The total cell counts were 611 and 1465 in the 100-μm- and 500-μm-deep channels, respectively. The reference value is the CD4 count measured using a C-Chip plastic hematocytometer chamber. The number of CD4 cells after spinning the cartridge is similar for both channels and is underestimated by approximately 14% compared with the reference value. This discrepancy could be caused by errors in estimating the channel volume or in analyzing cell images. We anticipate that the accuracy of the CD4 cell count can be improved by more precise fabrication of the helical minichannel and optimized parameters for image processing and analysis.

Importantly, confinement of the CD4 cells by spinning enhanced the accuracy of the CD4 cell count by factors of 2.1 and 5.8 for 100-µm- and 500-µm-deep channels, respectively. The precision of the CD4 cell counts was evaluated with the coefficient of variation, which were 4% and 2.6% for 100-μm- and 500-μm-deep channels, respectively. Enrichment of the CD4 cells by spinning improved the precision by a factor of 1.5 for the 500-µm-deep channel.

**Figure 10:**
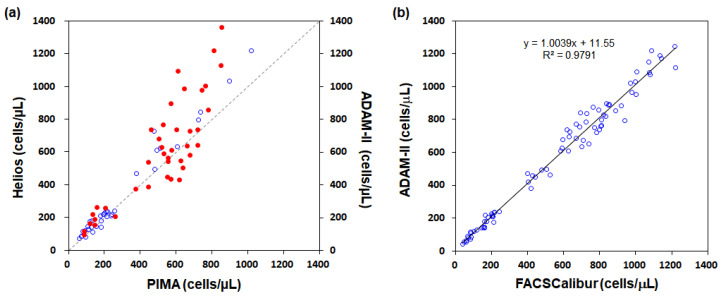
Comparison of CD4+ cell counts in diluted blood samples from human volunteers. (**a**) Helios and ADAM-II versus PIMA; (**b**) ADAM-II versus FACSCalibur.

For validation of the CD4 cell count measured with our system (Helios), we have compared our measurement with the commercially available CD4 cell counter, PIMA. Blood samples with low concentration CD4 cells that mimic blood from HIV/AIDS patients were made by diluting blood with phosphate buffered solution. Comparisons between the PIMA and Helios instruments shown in Figure 10a yielded a correlation with an *R*^2^ = 0.744. Compared with the large deviation at high-count group, middle- and low-count groups have higher correlations. In our previous study [24], we used the FACSCalibur flow cytometer (BD Biosciences, San Jose, CA, USA) as a gold standard to validate a benchtop fluorescence cell counter ADAM-II (NanoEnTek, Seoul, South Korea) as shown in Figure 10b and the ADAM-II was then compared with the PIMA as shown in Figure 10a. Therefore, we can make an indirect comparison between the FACSCalibur and the PIMA and between the FACSCalibur and the Helios. With known correlations among the FACSCalibur, the ADAM-II and the PIMA, we can determine the correlation between the FACSCalibur and the PIMA to be as high as (*R*_FA_)(*R*_AP_) + [(1 − *R*_FA_^2^)(1 − *R*_AP_^2^)]^1/2^ or as little as (*R*_FA_)(*R*_AP_) − [(1 − *R*_FA_^2^)(1 − *R*_AP_^2^)]^1/2^, but it cannot be outside this range of 0.912–0.99. This *R*^2^ value indicates that the correlation is reasonably high and the counting results agree. Here, *R*_FA_ represents the *R*^2^ value between the FACSCalibur and the ADAM-II and *R*_AP_ indicates the *R*^2^ value between the ADAM-II and the PIMA.

## 4. Discussion

HIV can weaken the immune system of humans and cause AIDS. Although a person infected with HIV can live only for 9–11 years, the progress of HIV can be controlled with proper treatment after early diagnosis. HIV/AIDS is one of the major causes of death in resource-limited regions, where diagnostic devices for detecting the disease need to be rapid, simple, portable and affordable [2]. Existing devices mostly use a 2D flat chamber and can only accommodate a very small sample volume. Furthermore, the 2D flat chamber requires a stage that translates in both the *x*- and *y*-directions to capture all areas of the chamber. As a result, the equipment size is large and complex software is required.

Our helical minichannel-based cell counting system overcomes these issues by employing a single DC motor to spin the sample cartridge, obviating the need for a translational stage. The system is controlled remotely using a smartphone over a Bluetooth connection, and its detection efficiency can be increased by confining cells to the top of the channel under the effect of centrifugal force by increasing the spin speed of the cartridge. We confirmed that cells with weak fluorescence intensity, such as fluorescently-labeled CD-expressing cells, can be concentrated within the focal plane, enabling detection without implementing autofocusing. Therefore, the additional cost of increasing the sensitivity is saved and the image acquisition rate can be increased. A sample preparation function can be added in future, as demonstrated by many research groups, for separation of particles based on size, detection of pathogenic bacteria and isolation of leukocytes from whole blood with the aid of spiral or helical microchannels [25,26,27]. Seminal works on separating and sorting cells in microfluidic chips include the use of acoustic or magnetic forces, such as bulk acoustic traveling waves for manipulating the position of the flowing particles [28] and the magnetographic array for capturing and enumerating single cells [29]. This system has a high correlation of counting with a gold standard flow cytometer. However, the required 1 inch × 1 inch microchip fabrication may be a little complex and expensive for commercialization. The helical minichannel-based sample cartridge has the advantage over other systems that it is made from a plastic material and can be mass produced at low cost.

Our ultimate goal is to develop a helical minichannel-based rapid POC diagnostic system that enumerates CD4 T-cells with high accuracy and precision. To conform to the ASSURED criteria (affordable, sensitive, specific, user friendly, rapid and robust, equipment free and deliverable to end users) set out by the WHO for microfluidic POC devices [15], we aim to integrate and automate all the necessary functional modules in a mobile platform for sample loading, reagent mixing, transport, detection and analysis by adopting a single DC motor that controls the rotation of the sample cartridge in various prescribed modes. We have implemented sample mixing inside the cartridge through bidirectional rotation of the cartridge by motor control and enhanced the efficiency of antigen–antibody reaction without lysing a finger-pricked whole blood sample [30]. The reacted sample can be transported from the mixing chamber to the helical minichannel by rotating the motor at lower speeds in one direction, which generates a pressure inside the reaction chamber based on the nut and bolt mechanism.

In the present study, we developed a method to enhance the detection efficiency by confining and enriching cells in the focal plane along the helical minichannel immediately before the detection step with fast spinning of the sample cartridge, rather than limiting the channel depth and sample volume as is done in conventional POC diagnostic devices. Confinement was highly effective in counting weakly fluorescent and low concentration cells such as CD4+ T-cells, improving the accuracy and precision greatly. If combined with a sample reaction module, we anticipate that a fully integrated POC system for counting various CD-expressing cells can be implemented successfully in near future. The integrated and automated system would provide a cost-effective and rapid solution for POC testing compared to existing technologies and also minimize human intervention, reducing laborious tasks and sample contamination.

## 5. Conclusions

Confinement and enrichment of microparticles were investigated in stationary and spinning sample cartridges having a helical minichannel filled with 10% fluorescent beads and a 10% glycerol mixture, which has the same density as blood plasma. It was found that when the channel was stationary, no particle confinement was achieved; however, when it was spun at 1000–3000 rpm for 10–120 s, particles were observed to be confined to the top of the channel, facilitating detection of the enriched particles within the focal depth. We demonstrated the effect of spinning the cartridge using centrifugal force on particle confinement. Moreover, the forces acting on the particles were determined and the velocity and time required for the particles to move toward the top of the channel under centrifugal force were verified against the theoretical and the experimental values. Based on the results of fluorescent bead confinement, we applied the strategy to enumerate low concentration CD4 cells with weak fluorescence intensity. Since the weakly fluorescent CD4 cells were detected through confinement and enrichment even in the channel with a 500-μm depth, a large sample volume can be analyzed, thereby improving the accuracy of CD4 cell count by a factor of 5.8 and enhancing the precision by a factor of 1.5 with a coefficient of variation of 2.6%.
